# Tremor-Dominant in Parkinson Disease: The Relevance to Iron Metabolism and Inflammation

**DOI:** 10.3389/fnins.2019.00255

**Published:** 2019-03-27

**Authors:** Teng-Hong Lian, Peng Guo, Li-Jun Zuo, Yang Hu, Shu-Yang Yu, Qiu-Jin Yu, Zhao Jin, Rui-Dan Wang, Li-Xia Li, Wei Zhang

**Affiliations:** ^1^Department of Neurology, Beijing Tiantan Hospital, Capital Medical University, Beijing, China; ^2^Department of Internal Medicine, Beijing Tiantan Hospital, Capital Medical University, Beijing, China; ^3^Center for Cognitive Neurology, Department of Neurology, Beijing Tiantan Hospital, Capital Medical University, Beijing, China; ^4^China National Clinical Research Center for Neurological Diseases, Beijing, China; ^5^Key Laboratory for Neurodegenerative Disorders of the Ministry of Education, Capital Medical University, Beijing, China; ^6^Center of Parkinson’s Disease, Beijing Institute for Brain Disorders, Beijing, China; ^7^Beijing Key Laboratory on Parkinson’s Disease, Beijing, China

**Keywords:** Parkinson disease, tremor-dominant, iron metabolism, inflammation, IL-6

## Abstract

**Background:** Tremor is one of the most predominant symptoms of patients with Parkinson disease (PD), but the underlying mechanisms for tremor relating to iron and its metabolism-related proteins and the inflammatory factors in cerebrospinal fluid (CSF) and serum have not been fully elucidated.

**Methods:** A total of 135 PD patients were divided into a tremor-dominant (PD-TD) group (*N* = 74) and a postural instability and gait difficulty-dominant (PD-PIGD) group (*N* = 39) based on the ratio of mean tremor score to the mean bradykinesia/rigid score of the Unified Parkinson’s Disease Rating Scale (UPDRS) III. Age and sex-matched healthy controls were recruited (*N* = 35). Demographic variables were evaluated; iron and its metabolism-related proteins and the inflammatory mediators in both CSF and serum were measured in these groups. The relevance of iron metabolism, inflammation and PD-TD were analyzed.

**Results:** (1) The PD-TD group had significantly decreased L-ferritin, increased iron levels in CSF and increased ferritin levels in the serum compared with the PD-PIGD and control groups (*P* < 0.05). (2) The PD-TD group had significantly enhanced IL-6 levels in both CSF and serum compared with the PD-PIGD and control groups (*P* < 0.05). (3) In CSF, the IL-6 level was increased as the iron level was elevated in the PD-TD group (*r* = 0.308, *P* = 0.022). In serum, the IL-6 level was increased as the ferritin level was elevated in the PD-TD group (*r* = 0.410, *P* = 0.004).

**Conclusion:** The interplay between disturbed iron metabolism and relevant inflammation might modulate clinical phenotypes of PD.

## Introduction

Parkinson disease (PD) is a progressive neurodegenerative disease characterized by classical motor manifestations, including resting tremor, bradykinesia, rigidity, and gait and postural abnormalities. Based on these symptoms, PD can be divided into three motor phenotypes: tremor-dominant (PD-TD), postural instability and gait difficulty-dominant (PD-PIGD), and a mixed group (Isaias et al., 2011). It was reported that patients with PD-TD tend to have slower disease progression (Moretti et al., 2017), fewer non-motor symptoms (Ba et al., 2016), more chances of improvement when using levodopa and higher survival rates (Rajput et al., 2017) compared to patients with PD-PIGD.

The typical motor symptoms of PD are predominantly attributed to the neurodegeneration of dopamine-producing neurons in the substantia nigra (SN) and the dysfunction of the basal ganglia (Shulman et al., 2011; De Virgilio et al., 2016). Tremor and non-tremor phenotypes have distinct underlying pathophysiologic mechanisms in PD (Thenganatt and Jankovic, 2014). It is believed that the increased interactions between the basal ganglia and the cerebello-thalamo-cortical circuit may contribute to parkinsonian tremor (Helmich et al., 2011; Helmich, 2018). Moreover, the dopamine depletion in the globus pallidus is associated with the severity of a clinical tremor (Helmich et al., 2011). The tremor-associated drive from the subthalamic nucleus (STN) to the muscles has been confirmed, so STN may also be directly involved in tremor generation (Guan et al., 2017). However, as one of the supporting characteristics of PD, the underlying mechanisms for tremor have not been fully elucidated.

Abnormal iron accumulation is associated with a host of neurodegenerative diseases, such as PD and Alzheimer disease, via an abnormal deposition in specific brain regions or a malfunction of iron homeostasis (Sian-Hulsmann et al., 2011). Most of the research in PD patients focuses on the brain iron overload in regions such as the SN, putamen and globus pallidus (Sian-Hulsmann et al., 2011; Liu et al., 2017; Sjostrom et al., 2017; Xuan et al., 2017; Yu et al., 2018). A study of magnetic susceptibility weighted imaging show that the iron depositions in the dentate nucleus of the cerebellum and the red nucleus, are correlated with tremor in PD (Guan et al., 2017). However, few experiments have been conducted on iron and its metabolism-related proteins in both the central and peripheral systems, within the different motor phenotypes of PD.

A growing set of observations from post-mortem and *in vivo* studies suggest that inflammation in both the central and peripheral systems may be one of the primary mechanisms involved in neuronal degeneration in PD (Hirsch and Hunot, 2009). Inflammation in SN may serve as a driving force of neuronal death (Block et al., 2007) and the active peripheral inflammation can promote dopaminergic cell loss by aggravating and synergizing central inflammatory responses (Tiwari and Pal, 2017). Moreover, inflammation is likely to cause iron deposition by inducing changes in iron metabolism proteins (Urrutia et al., 2013) and inhibiting the mitochondrial complex I (Urrutia et al., 2014). In return, excessive free iron induces cellular destruction and neurodegeneration via oxidative and nitrative stress, inflammation, and excitotoxicity (Sian-Hulsmann et al., 2011). The relevance of abnormal iron metabolism and related inflammation has been found in some parkinsonian non-motor symptoms, like pure apathy (Wang et al., 2016), sleep disorders (Yu et al., 2013), and especially rapid eye movement sleep behavior disorder (Hu et al., 2015). The ratio of neutrophil-lymphocyte, a marker of inflammation, has been observed to have a positive association with motor severity in the PD-TD group (Sanjari Moghaddam et al., 2018). Yet, the role among parkinsonian tremor, abnormal iron metabolism, and inflammation is seldom investigated.

In this study, the levels of iron and the proteins related to iron metabolism, including ferritin, *H*-ferritin and L-ferritin, transferrin, and lactoferrin, and inflammatory mediators, including interleukin (IL)-6, IL-1β, prostaglandin (PG) E_2_, nitric oxide (NO), and hydrogen peroxide (H_2_O_2_), were detected in both the CSF and serum in the PD-TD, PD-PIGD, and the healthy control groups. We then analyzed the relationship between iron and the proteins related to iron metabolism and inflammatory mediators in the CSF and serum of PD-TD patients.

## Materials and Methods

### Subjects

#### PD Patients

A total of 135 PD patients were consecutively recruited from the Department of Neurology, Beijing Tiantan Hospital, Capital Medical University. All PD patients satisfied the Movement Disorder Society Clinical Diagnostic Criteria for Parkinson disease (Hughes et al., 1992).

Demographic variables, including sex, age, education level, disease duration, age at disease onset, vascular risk factors, like hypertension, diabetes, hyperlipidemia and smoking, the number and levodopa equivalent daily dose (LEDD) of patients under dopaminergic treatment for PD were recorded. The severity was evaluated using the Hohen-Yahr stage.

PD patients with systemic diseases, especially infectious and autoimmune diseases and hematological diseases, and patients who have experienced blood loss were excluded.

#### Control Subjects

Normal controls were required to satisfy the following criteria: no intracranial diseases, no neurological symptoms or signs and normal magnetic resonance imaging results of the head. The other exclusion criteria for the control subjects were the same as those for the PD patients. Finally, 35 age and sex-matched healthy controls were recruited for this study.

### Classification of Motor Phenotypes for PD Patients

Motor symptoms were assessed by the Unified Parkinson’s Disease Rating Scale (UPDRS) III. Motor phenotypes were identified based on the ratio of mean tremor score (sum of items 20 and 21 in UPDRS III divided by four) to the mean bradykinesia/rigid score (sum of items 22–27 and 31 in UPDRS III divided by 15). Patients with a ratio greater than 1.0, less than 0.80, and between 0.80 and 1.0 were classified into PD-TD, PD-PIGD, and mixed-types (Schiess et al., 2000), respectively. The patients of mixed-type were excluded due to the mixed motor phenotype.

### Collections of CSF and Serum

In total, 3 ml CSF and 2 ml venous whole blood was collected in polypropylene tubes at 07:00 to 10:00 in the fasting condition, with anti-parkinsonian medication withdrawal for 12–14 h if patients’ condition permitted (Zuo et al., 2016). CSF and serum samples were centrifuged immediately at 3000 rpm at 4°C. Approximately 0.5 ml volume of the supernatant of CSF and serum were put into separate Nunc cryotubes and frozen at -80°C. Freezing and thawing and protein degradation were carefully avoided.

### Detections of the Levels of Iron and Its Metabolism-Related Proteins in CSF and Serum

The levels of iron and ferritin, H-ferritin and L-ferritin, transferrin, and lactoferrin in the CSF and serum were measured by using the Enzyme-Linked Immunosorbent Assay (ELISA) (Piao et al., 2017). We performed two measurements and took the average of the two results for each patient. Ab83366 kit, Ab108837 kit, and Ab108911 kits (Abcam Company, Cambridge, United Kingdom) were used for iron, ferritin, and transferrin, respectively. The E01L0224 kit (Shanghai Lanji Biological Limited Company, Shanghai, China) was used for lactoferrin.

### Detections of the Levels of Inflammatory Factors in CSF and Serum

The levels of inflammatory factors, including IL-6, IL-1β, and PGE_2_ in the CSF and serum were measured using ELISA (Hu et al., 2015). A 1R040 kit (RB Company, Shanghai, China) was used for IL-1β. A human IL-6 ELISA kit (Beijing DOP Biotechnology Co., Ltd, Beijing, China) was used for IL-6. A CSB-E07965h kit (CUSABIO Company, Wuhan, China) was used for PGE_2_. The levels of free radicals, including NO and H_2_O_2_, were measured using the chemical colorimetric method. An A012 kit and A064 kit (Nanjing Jiancheng Biological Engineering Research Institute, Nanjing, China) were used for NO and H_2_O_2_, respectively.

### Data Analyses

Statistical analyses were conducted using SPSS Statistics 20.0 software (IBM Corporation, Armonk, New York, United States).

The normally distributed continuous variables were compared using a two-tailed *t*-test. The abnormally distributed continuous variables were compared using a non-parametric test. Discrete variables were compared using a Chi-square test. A *P*-value of less than an alpha level of 0.05 was defined as statistically significant.

Demographic variables were compared between the PD-TD and PD-PIGD groups. Multiple comparisons of the levels of iron and its metabolism-related proteins and inflammatory factors in the CSF and serum were compared among the control, PD-PIGD, and the PD-TD groups by using the Kruskal–Wallis test. A Bonferroni correction was made in the comparison of multiple inflammatory factors or iron/ferritin among the control, PD-PIGD and the PD-TD groups to control the family wise error rate. In order to adjust for the multiple tests, we set the significance to 0.05/6 in the comparison of the levels of iron and its metabolism-related proteins in the CSF or serum, and we set the significance to 0.05/5 in the comparison of the levels of inflammatory factors in the CSF or serum. *Post hoc* analyses were made by using the Bonferroni correction. We set the significance to 0.05/3 in the pair-wise of each group.

The Spearman correlation was used to test the association between iron and its metabolism-related proteins and inflammatory factors in the PD-TD group.

## Results

### Demographic Variables and Clinical Characteristics of PD-TD and PD-PIGD Groups

Among the 135 PD patients, 74 cases (54.81%) and 39 cases (28.89%) were classified into the PD-TD group and the PD-PIGD group, respectively.

Demographic variables, including sex, age, education level, age at disease onset, disease duration, vascular risk factors like hypertension, diabetes, hyperlipidemia and smoking, LEDD, and the number of patients under dopaminergic treatment in the PD-PIGD and PD-TD groups were compared (Table 1). The results demonstrated no significant differences in the above demographic variables between the two groups. More analyses of the correlation of the demographic variables with the following iron metabolism/inflammation markers can be seen in Supplementary Tables 1, 2.

**Table 1:** Clinical variables of PD-PIGD and PD-TD groups.

	PD-PIGD group	PD-TD group	*P*-value
	(39 cases )	(74 cases)	
Male (n, %)	21 (54.9 %)	41 (57.1 %)	0.874
Age (years, mean ± SD)	60.0 ± 9.5	58.8 ± 9.7	0.261
Low education level (less than 9 years) (n, %)	27 (62.7%)	46 (67.9%)	0.455
Age of disease onset (years, mean ± SD)	57.9 ± 9.5	55.0 ± 10.7	0.173
Disease duration (years, mean ± SD)	3.1 ± 2.7	3.9 ± 4.0	0.599
Diabetes (*n*, %)	3 (7.7%)	5 (6.8%)	1.000
Hypertension (*n*, %)	3 (7.7)	6 (8.1)	1.0000
Hyperlipidemia (*n*, %)	1 (2.6%)	0 (0.0%)	0.345
Smoking (*n*, %)	5 (12.8%)	8 (10.8%)	0.763
The number of patients under dopaminergic treatment (*n*, %)	13 (38.2%)	34 (46.0%)	0.583
LEDD for the patients under dopaminergic treatment (mg, mean ± SD)	47.3 ± 98.2 (13 cases)	57.3 ± 127.0 (34 cases)	0.764
Hoehn- Yahr stage (*n*, %)			
Early stage (stage 1–2.5)	31 (88.2%)	64 (84.5%)	0.427
Middle and late stage (stage 3–5)	8 (11.8%)	11 (15.5%)	
UPDRS III (points, mean ± SD)	30.5 ± 15.7	25.7 ± 16.4	0.047*


*^∗^*P* < 0.05.*

The PD-TD and PD-PIGD groups showed no significant difference in Hoehn-Yahr stage (Table 1), suggesting that the two groups did not differ in PD severity.

The UPDRS III score was significantly lower in the PD-TD group than in the PD-PIGD group (Table 1), indicating that the PD-TD group had significantly less severe motor symptoms than the PD-PIGD group.

### The Comparation of Iron and Iron Metabolism-Related Protein Between PD-TD and PD-PIGD Groups in Both Central and Peripheral Systems

The levels of iron, ferritin, *H*-ferritin, L-ferritin, transferrin, and lactoferrin in the CSF and serum were compared between the control, PD-PIGD and PD-TD groups (Table 2). Further comparation of the iron and its metabolism-related protein between the PD-PIGD and PD-TD groups of drug-naive patients can be seen in Supplementary Table 3.

**Table 2:** The levels of iron and its metabolism-related proteins in CSF and serum from the control, PD-PIGD and PD-TD groups.

	Control group	PD-PIGD group	PD-TD group	*P*	*P*^1^	*P*^2^	*P*^3^
	(35 cases)	(39 cases)	(74 cases)				
**CSF**							
Iron (nmol/ml, mean ± SD)	0.478 ± 0.307	1.179 ± 1.390	3.197 ± 5.261	<.**001**	0.026	<.**001**	**0**.**015**
Ferritin (ng/ml, mean ± SD)	7.337 ± 14.110	8.642 ± 8.527	5.587 ± 5.915	**0**.**002**	**0**.**004**	**0**.**001**	0.301
*H*-ferritin (ng/ml, mean ± SD)	2.142 ± 0.730	1.503 ± 0.850	1.146 ± 0.662	<.**001**	**0**.**007**	<.**001**	0.029
L-ferritin (ng/ml, mean ± SD)	2.496 ± 1.024	1.591 ± 0.618	1.261 ± 0.610	<.**001**	**0**.**008**	<.**001**	**0**.**016**
Transferrin (nmol/l, mean ± SD)	0.067 ± 0.020	0.146 ± 0.093	0.155 ± 0.083	**0**.**001**	**0**.**002**	**0**.**004**	0.490
Lactoferrin (ug/ml, mean ± SD)	67.445 ± 108.506	126.119 ± 68.233	127.016 ± 75.564	<.**001**	<.**001**	<.**001**	0.877
**Serum**
Iron (nmol/m, mean ± SD)	3.664 ± 1.490	2.688 ± 1.644	3.594 ± 1.541	**0**.**022**	**0**.**010**	0.735	0.020
Ferritin (ng/ml, mean ± SD)	16.053 ± 17.233	25.666 ± 19.928	59.521 ± 50.849	<.**001**	0.034	<.**001**	**0**.**006**
H-ferritin (ng/ml, mean ± SD)	2.211 ± 0.932	2.1200 ± 0.998	2.263 ± 0.881	0.754			
L-ferritin (ng/ml, mean ± SD)	2.868 ± 0.813	2.202 ± 0.794	2.572 ± 1.014	0.145			
Transferrin (nmol/l, mean ± SD)	3.582 ± 1.900	0.247 ± 0.644	3.005 ± 13.427	<.**001**	<.**001**	<.**001**	0.022
Lactoferrin (ug/ml, mean ± SD)	97.622 ± 106.311	139.950 ± 82.928	161.260 ± 74.805	<.001**	**0**.**004**	<.**001**	0.186


**P*, Kruskal–Wallis test among control, PD-PIGD and PD-TD groups, α = 0.008; *P*^*1*^, PD-PIGD group vs. Control group, α = 0.017; *P*^*2*^, PD-TD group vs. Control group, α = 0.017; *P*^*3*^, PD-TD group vs. PD-PIGD group, α = 0.017. The bold values are the *p*-values less than α.*

In the CSF, iron levels in the PD-TD group were significantly higher than those in both the control and PD-PIGD groups (*P* < 0.001, *P* = 0.045, respectively). L-ferritin levels in the PD-TD group were prominently lower than those in the control and PD-PIGD groups (*P* < 0.001, *P* = 0.019, respectively). These results were consistent with the comparison of the above factors between the PD-PIGD and PD-TD groups of the drug-naive patients (Supplementary Table 3).

In the serum, ferritin level in the PD-TD group was strikingly lower than those in the PD-PIGD and control groups (*P* < 0.001, *P* = 0.019, respectively).

### The Comparation of Inflammatory Factors Between PD-TD and PD-PIGD Groups in Both Central and Peripheral Systems

The levels of inflammatory factors, including IL-1β, IL-6, PGE_2_, NO, and H_2_O_2_ in the CSF and serum were compared among the control, PD-PIGD and PD-TD groups (Table 3). Further comparison of the inflammatory factors between the PD-PIGD and PD-TD groups of drug-naive patients could be seen in Supplementary Table 4.

**Table 3:** The levels of inflammatory factors in CSF and serum from the control, PD-PIGD and PD-TD groups.

	Control group	PD-PIGD group	PD-TD group	*P*	*P*^1^	*P*^2^	*P*^3^
	(35 cases)	(39 cases)	(74 cases)				
**CSF**							
IL-1β (pg/ml, mean ± SD)	18.608 ± 16.714	19.342 ± 8.767	17.435 ± 12.566	0.083			
IL-6 (pg/ml, mean ± SD)	1.414 ± 0.295	2.410 ± 1.884	3.656 ± 2.098	<.**001**	0.017	<.**001**	**0**.**007**
PGE_2_ (pg/ml, mean ± SD)	15.514 ± 16.394	12.090 ± 5.481	9.529 ± 5.257	0.085			
H_2_O_2_ (mmol/L, mean ± SD)	4.497 ± 7.148	7.101 ± 6.947	8.645 ± 7.830	**0**.**004**	**0**.**015**	**0**.**001**	0.490
NO (mmol/L, mean ± SD)	52.224 ± 30.709	75.648 ± 40.803	57.724 ± 31.411	**0**.**025**	**0**.**016**	0.021	0.485
**serum**							
IL-1β (pg/ml, mean ± SD)	17.738 ± 23.475	18.720 ± 12.859	17.778 ± 12.821	<.**001**	<.**001**	<.**001**	0.567
IL-6 (pg/ml, mean ± SD)	1.478 ± 0.243	3.206 ± 2.526	11.944 ± 29.084	<.**001**	0.071	<.**001**	**0**.**004**
PGE_2_ (pg/ml, mean ± SD)	11.029 ± 7.953	8.220 ± 4.855	11.158 ± 12.048	0.401			
H_2_O_2_ (mmol/L, mean ± SD)	28.040 ± 14.489	39.356 ± 22.611	39.627 ± 29.175	**0**.**034**	0.037	**0**.**014**	0.936
NO (mmol/L, mean ± SD)	58.317 ± 53.023	56.610 ± 34.381	55.356 ± 27.895	0.375			


**P*, Kruskal–Wallis test among control, PD-PIGD and PD-TD groups, α = 0.008; *P*^*1*^, PD-PIGD group vs. Control group, α = 0.017; *P*^*2*^, PD-TD group vs. Control group, α = 0.017; *P*^*3*^, PD-TD group vs. PD-PIGD group, α = 0.017. The bold values are the *p*-values less than α.*

In the CSF, the PD-TD group had significantly enhanced IL-6 levels compared with the PD-PIGD and control groups (*P* < 0.001, *P* = 0.020, respectively). In the serum, IL-6 levels in the PD-TD group were strikingly increased compared with the PD-PIGD and control groups (*P* < 0.001, *P* = 0.012, respectively). These results were consistent with the comparison of the inflammatory factors between PD-PIGD and PD-TD groups of the drug-naive patients (Supplementary Table 4).

Further analysis suggested that IL-6 level in the CSF had a positive and significant correlation with IL-6 levels in the serum (*r* = 0.257, *P* = 0.022).

### The Relationship Between Iron Metabolism and Inflammationin PD-TD Patients

In the CSF, we observed that the IL-6 levels increased alongside elevated iron levels in the PD-TD group (*r* = 0.308, *P* = 0.022). In the serum, we found that the IL-6 levels increased alongside enhanced ferritin levels in the PD-TD group (*r* = 0.410, *P* = 0.004).

## Discussion

In this study, 62.22% of all cases were classified as PD-TD, indicating that tremor is one of the most predominant symptoms of PD. However, the PD-TD group displayed less severe motor symptoms (Table 1) indicated by a lower UPDRS III score than the PD-PIGD group, consistent with a previous longitudinal study (Rajput et al., 2017). More widespread biochemical abnormalities in the brain may explain the more severe motor symptoms of PD-PIGD patients (Rajput et al., 2008).

Iron plays an essential role on many biological activities, including DNA synthesis, oxygen transport and mitochondrial respiration (Sian-Hulsmann et al., 2011). However, elevated iron levels can elicit a cascade of cellular deleterious events (Munoz and Humeres, 2012). The consequence of the dysregulation of iron and its metabolism may be the primary cause of PD (Weinreb et al., 2013).

In the PD-TD group, iron levels in the CSF were strikingly increased compared with the PD-PIGD and control group (Table 2), indicating that excessive iron in the brain might be associated with PD-TD. Furthermore, the role of the proteins involved in iron metabolism was investigated. As it is known, ferritin, the most common iron storage protein in the brain, can reduce the toxic effects of excessive iron (Henle et al., 1996; Zhang et al., 2014). Ferritin comprises of heavy-ferritin (*H*-ferritin) and light-ferritin (L-ferritin). *H*-ferritin converts toxic Fe (II) into less toxic Fe (III) and participates in the absorption and utilization of iron (Harrison and Arosio, 1996). In contrast, L-ferritin contributes to the long-term safe storage of iron (Harrison and Arosio, 1996) and is predominantly expressed in microglia (Connor et al., 2001), which are mostly scavenger cells. In this study, compared with the PD-PIGD and control groups, L-ferritin levels in the CSF in PD-TD group were especially decreased (Table 2). In summary, abnormal iron metabolism, especially the decrease of L-ferritin levels, and subsequent excessive iron deposition in the brain, may be related to tremor in PD patients.

The plasma ferritin level was strongly increased in the PD-TD group (Table 2). Results of plasma ferritin changes in PD patients are inconsistent (Madenci et al., 2012; Costa-Mallen et al., 2015). Ferritin levels in the serum can be increased with systemic iron overloaded (Kuhn, 2015), but we failed to identify excessive iron in the serum of the PD-TD group. We speculate that the plasma ferritin level may be increased in two ways. On the one hand, PD-TD patients may have a damaged blood-brain barrier, allowing ferritin transport from the CSF to the plasma and inducing an elevation of ferritin in the plasma. Though the PD-TD group had a lower L-ferritin level in the brain, there was no significant difference in the ferritin level between the PD-TD and PD-PIGD groups (Table 2). On the other hand, it was found that plasma ferritin can also be increased with body iron deficiency, in the inflammatory condition, due to iron retention in macrophages (Cohen et al., 2010). Additionally, it was demonstrated that the ferritin level was positively correlated with the IL-6 level in the serum of PD-TD patients. Therefore, we speculate that abnormal peripheral iron metabolism may also play a role on PD-TD due to more severe inflammation in the serum.

Peripheral iron can be transferred across the blood–brain barrier via transferrin, lactoferrin and their receptor, as well as divalent cation metal transporters (Ponka, 2004). In this study, compared with the control group, iron and transferrin levels were increased in the CSF, but decreased in the serum, in PD patients (Table 2). We hypothesize that these changes may be the result of the abnormal transfer of transferrin from the peripheral to the brain, further leading to the abnormal deposition of iron in PD brains. Though an imbalance of transferrin in the peripheral and central systems was seen in PD patients, we failed to see differences in transferrin and lactoferrin levels in the CSF and serum between the PD-TD and the PD-PIGD groups (Table 2). More studies are needed to explore whether iron depositions in the brains of PD-TD patients are resulted from the transfer of iron from the periphery to the central nervous system.

Accumulating evidence reveals a pivotal role of chronic neuroinflammation on the pathologic features of PD(Collins et al., 2012). Inflammatory mediators, such as NO, TNF-α, IL-1β, and IL-6 may contribute to the progression of PD (Niranjan et al., 2012; Williams-Gray et al., 2016; Kouchaki et al., 2018). This study showed that the PD-TD group had a significantly elevated IL-6 level in the CSF when compared with the PD-PIGD and control groups (Table 2). As it is known, IL-6 is a pleiotropic cytokine involved in the acute phase response. It can trigger degeneration and even the death of neurons after injury and infection in both the peripheral and central nervous system (Gadient and Otten, 1997; Jones and Jenkins, 2018). A *de novo* PD patient has significantly elevated IL-6 levels in the CSF when compared to the control group (Blum-Degen et al., 1995). Moreover, it has been demonstrated that men with elevated IL-6 levels in the serum had a higher risk of PD, but the same influence was not seen in other inflammatory biomarkers, like C-reactive protein, fibrinogen, and TNF-α (Chen et al., 2008). Moreover, a positive correlation has been found between plasma IL-6 levels and the UPDRS III score (Delgado-Alvarado et al., 2017). Some studies fail to certify the same difference of the plasma IL-6 level between PD and control groups and the inconsistency may be related to the recruitment of patients with a shorter disease duration than in the current study (Blum-Degen et al., 1995). Collectively, many existing studies have shown that increased IL-6 levels in both the brain and the serum may contribute to the progression of PD. However, to our knowledge, no studies have examined the role of IL-6 on parkinsonian tremor. This study is the first to reveal that inflammation, especially IL-6 in both the peripheral and central nervous system, may play a critical role on TD of PD patients. Moreover, the spearman correlation analysis further showed that IL-6 levels in the serum were significantly correlated with IL-6 levels in the CSF, indicating that peripheral inflammation exacerbates central inflammation in PD-TD patients.

Similar changes of iron accumulation and inflammation can also be seen in PD-TD and PD-PIGD groups of drug-naive patients, who do not have the following influence of anti-parkinsonian treatment. Different clinical symptoms of PD have a different response to anti-parkinsonian drugs. For example, bradykinesia generally responds very well to levodopa (Nutt et al., 2011), while tremor does not (Yahr et al., 1969). Anti-parkinsonian drugs may produce effects on inflammatory factors, like IL-6, IL-8 and NO (Parrado et al., 2012; Wang et al., 2019). For example, it has been found that DA alone does not regulate the inflammatory factors directly, like IL-6, TNF-α or NO, but it inhibits lipopolysaccharide-induced NO production in microglial cells. In our study, anti-parkinsonian drugs were withdrawn at least 12–14 h before obtaining the CSF and serum samples. Thus, the anti-parkinsonian treatment might have little influence on the results. Therefore, the changes of iron/ inflammation makers of the two groups remain the same in both total PD patients and the drug-naive PD patients.

The interplay between iron accumulation and inflammation in the neurodegenerative process may lead to cell death in PD (Urrutia et al., 2014). Here, positive correlations were found between the IL-6 level and iron level in the CSF and ferritin level in the serum in PD-TD patients, indicating that increased iron depositions in the brain and systemic abnormal iron metabolism worsened as neuroinflammation intensified. As a cross-sectional study in humans, this investigation could not reveal whether abnormal iron metabolism was the cause or the consequence. However, the vicious cycle of inflammation and iron-induced oxidative damage in both the peripheral and central nervous systems might be given considerable weight in contributing to PD-TD. As known, the basal ganglia nuclei are transiently activated at the onset of tremor episodes, whereas activity in the cerebello-thalamo-cortical circuit fluctuates with tremor amplitude (Helmich et al., 2011) in PD patients. Iron depositions in the dentate nucleus of the cerebellum, red nucleus, and the subcortical nuclei in the cerebello-thalamo-cortical circuit, have been reported to be correlated with tremor in PD (Guan et al., 2017). Moreover, patients with the mutation causing abnormal ferritin metabolism and iron deposition in the globus pallidus presented with symptoms of extrapyramidal dysfunction including tremor (Ponka, 2002). In conclusion, overloaded iron might be involved in the presence of tremor of PD. We put forward the hypothesis that insufficient L-ferritin is unable to handle the overload iron, leading to iron deposition in the brain. Iron deposition and related inflammation are part of a synergistic self-propelling cycle, and result in neuronal death in TD-associated regions, like the basal ganglia and the cerebello-thalamo-cortical circuit, contributing to tremor in PD. However, the role of each inflammatory cytokine on different forms of PD has not been well acknowledged. It has been found that different cytokines may be involved in the pathophysiology of specific motor and non-motor symptoms of PD (Menza et al., 2010; Hu et al., 2015; Pereira et al., 2016). Some researchers postulate the hypothesis that inflammation may accompany PD progress and through the Braak stage, inflammation may move from the peripheral to the central nervous system to destroy different neurons, resulting in different forms of PD (Barnum and Tansey, 2012). Therefore, longitudinal studies are necessary in order to evaluate the impact of inflammatory factors on the motor and non-motor progression in PD.

## Conclusion

TD-PD patients exhibit decreased L-ferritin levels and subsequent excessive iron deposition in the brain and increased ferritin levels in the serum, as well as inflammation in both the peripheral and central nervous systems. The interplay between disturbed iron metabolism and inflammation may cast a new light on the mechanism underlying tremor in PD.

This investigation had the following limitations. First, CSF samples were relatively insufficient in healthy controls since most of the subjects were old and some of them had spinal deformities and bone hyperplasia. A further analysis should include more healthy controls. Second, since PD-TD has a higher frequency than PD-PIGD, patients with PD-TD were more than those with PD-PIGD, as expected. The imbalanced sample size of the two groups may cause a potential bias. In the future, we will recruit PD patients to amplify the sample size, aiming to reduce the bias. Third, the altered levels of iron and its metabolism-related proteins in the serum and CSF from PD-TD patients did not indicate that the iron accumulated in a certain brain region, such as the substantia nigra, therefore, neuroimaging studies are much needed in a further study. Fourth, though most of the PD patients had a significant response to levodopa, we still cannot guarantee that none of them will be diagnosed with parkinsonism, like multiple system atrophy (MSA), 2–3 years later since MSA patients may present symptoms very similar to PD and also have very good response to levodopa within 2–3 years of symptom onset. Hence, 2–3 years of follow-ups is very important for the confirmation of diagnosis. We will perform a follow-up in a future study. Lastly, as a cross-sectional study, this study had limited grounds for drawing definite conclusions. Moreover, some iron metabolism-related proteins, including divalent metal transporter 1 and ceruloplasmin were not included in this investigation. Therefore, we will continue to collect more CSF samples from healthy controls and a longitudinal study is being considered to explore the correlation of iron and iron metabolism-related proteins with inflammatory factors in PD-TD patients.

## Data Availability

The datasets generated for this study are available on request to the corresponding author.

## Ethics Statement

This study was carried out in accordance with the recommendations of International Ethical Guidelines for Biomedical Research Involving Human Subjects, Council for International Organizations of Medical Sciences. The protocol was approved by the Ethics Committee of the Beijing Tiantan Hospital. All subjects gave written informed consent in accordance with the Declaration of Helsinki.

## Author Contributions

T-HL contributed to drafting the manuscript, study design, analysis of data, accepts responsibility for conduct of research and will give final approval, acquisition of data, and statistical analysis. PG contributed the study design, accepts responsibility for the conduct of research and will give final approval, and acquisition of data. L-JZ, YH, S-YY, Q-JY, ZJ, R-DW, and L-XL contributed to accepts responsibility for the conduct of research and will give final approval, acquisition of data. WZ contributed to the study design, analysis of the data, accepts responsibility for the conduct of research and will give final approval, acquisition of data, and statistical analysis.

## Conflict of Interest Statement

The authors declare that the research was conducted in the absence of any commercial or financial relationships that could be construed as a potential conflict of interest.
