# Supplementary material for: Tremor-Dominant in Parkinson Disease: The Relevance to Iron Metabolism and Inflammation
Source: Front Neurosci. 2019 Mar 27;13:255. doi: 10.3389/fnins.2019.00255 (PMC6445850; doi:10.3389/fnins.2019.00255)
Supplement: Supplementary file 1 [file Table_1.DOCX]

Supplementary Material

|  |  | Age | | Disease duration | | Age of disease onset | | LEDD | |
| --- | --- | --- | --- | --- | --- | --- | --- | --- | --- |
|  |  | r | P | r | P | r | P | r | P |
| **Serum** | Iron  [nmol/ml, median (quartile)] | 0.004 | 0.968 | 0.085 | 0.378 | -0.024 | 0.81 | 0.134 | 0.165 |
|  | Ferritin  [ng/ml, median (quartile)] | **0.248^**^** | 0.01 | 0.107 | 0.267 | 0.172 | 0.078 | 0.022 | 0.819 |
|  | H-ferritin  [ng/ml, median (quartile)] | -0.044 | 0.672 | 0.04 | 0.696 | -0.079 | 0.445 | -0.007 | 0.944 |
|  | L-ferritin  [ng/ml, median (quartile)] | 0.135 | 0.187 | 0.027 | 0.792 | 0.096 | 0.354 | 0.095 | 0.35 |
|  | Transferrin  [nmol/l, median (quartile)] | 0.01 | 0.92 | 0.004 | 0.967 | -0.017 | 0.859 | 0.019 | 0.842 |
|  | Lactoferrin  [ug/ml, median (quartile)] | 0.129 | 0.182 | 0.007 | 0.946 | 0.066 | 0.505 | -0.04 | 0.678 |
|  | IL-1β  [pg/ml, median(quartile)] | 0.041 | 0.673 | -0.052 | 0.591 | -0.005 | 0.962 | 0.032 | 0.743 |
|  | IL-6  [pg/ml, median(quartile)] | 0.065 | 0.575 | -0.034 | 0.763 | 0.021 | 0.852 | 0.012 | 0.92 |
|  | PGE_2_  [pg/ml, median(quartile)] | 0.006 | 0.95 | **-.194^*^** | 0.046 | 0.085 | 0.394 | -0.02 | 0.837 |
|  | H_2_O_2_  [mmol/L, median(quartile)] | -0.094 | 0.337 | -0.1 | 0.305 | -0.062 | 0.53 | 0.012 | 0.903 |
|  | NO  [mmol/L, median(quartile)] | -0.056 | 0.576 | -0.016 | 0.874 | -0.123 | 0.22 | -0.048 | 0.628 |
| **CSF** | Iron  [nmol/ml, median (quartile)] | 0.01 | 0.912 | 0.125 | 0.162 | -0.074 | 0.422 | 0.041 | 0.65 |
|  | Ferritin  [ng/ml, median (quartile)] | 0.102 | 0.254 | -0.125 | 0.16 | 0.171 | 0.059 | -0.093 | 0.297 |
|  | H-ferritin  [ng/ml, median (quartile)] | 0.135 | 0.172 | -0.17 | 0.084 | **0.221^*^** | 0.025 | -0.117 | 0.236 |
|  | L-ferritin  [ng/ml, median (quartile)] | -0.046 | 0.645 | **-0.227^*^** | 0.02 | 0.057 | 0.569 | -0.044 | 0.658 |
|  | Transferrin  [nmol/l, median (quartile)] | 0.081 | 0.365 | -0.056 | 0.527 | 0.146 | 0.109 | 0.028 | 0.754 |
|  | Lactoferrin  [ug/ml, median (quartile)] | -0.007 | 0.94 | -0.103 | 0.248 | 0.062 | 0.494 | -0.079 | 0.373 |
|  | IL-1β  [pg/ml, median(quartile)] | 0.058 | 0.516 | -0.001 | 0.991 | 0.049 | 0.597 | -0.058 | 0.517 |
|  | IL-6  [pg/ml, median(quartile)] | 0.07 | 0.51 | 0.101 | 0.34 | 0.029 | 0.783 | 0.154 | 0.144 |
|  | PGE_2_  [pg/ml, median(quartile)] | -0.118 | 0.185 | -0.033 | 0.715 | -0.106 | 0.246 | 0.033 | 0.708 |
|  | H_2_O_2_  [mmol/L, median(quartile)] | 0.114 | 0.2 | **0.186^*^** | 0.035 | 0.002 | 0.98 | -0.054 | 0.547 |
|  | NO  [mmol/L, median(quartile)] | 0.119 | 0.184 | -0.006 | 0.949 | 0.083 | 0.364 | -0.028 | 0.755 |

**Supplementary Table 1**. The correlation of the demographic variables and iron metabolism/inflammation markers in CSF and serum from control, PD-PIGD and PD-TD groups. r: correlation coefficient. **: P＜0.01, *: P＜0.05.

**Supplementary Table 2**. The levels of iron and its metabolism-related proteins, inflammatory factors in CSF and serum base on the demographic variables. **: P＜0.01, *: P＜0.05.

|  |  | Gender | | | Education level | | | Hoehn- Yahr stage | | |
| --- | --- | --- | --- | --- | --- | --- | --- | --- | --- | --- |
|  |  | Male | Female | P | Less than 9 years | Equal or greater than 9 years | P | Early stage | Middle and late stage | P |
| **Serum** | Iron  [nmol/ml, median (quartile)] | 2.976  (2.256, 4.683) | 2.615  (1.894, 4.736) | 0.374 | 2.976  (2.216, 4.822) | 2.783  (2.220, 4.608) | 0.472 | 2.987  (2.251, 4.879) | 2.545  (1.121, 3.475) | 0.056 |
|  | Ferritin  [ng/ml, median (quartile)] | 19.596  (10.726, 75.192) | 35.872  (15.431, 61.922) | 0.241 | 20.76  (11.774, 68.514) | 34.136  (11.225, 72.272) | 0.611 | 22.967  (10.706, 62.255) | 34.108  (17.197, 86.773) | 0.300 |
|  | H-ferritin  [ng/ml, median (quartile)] | 2.305  (1.567, 3.013) | 1.996  (1.364, 2.639) | 0.170 | 2.41  (1.614, 2.964) | 1.996  (1.441, 2.832) | 0.203 | 2.163  (1.408, 2.878) | 2.327  (1.929, 2.829) | 0.551 |
|  | L-ferritin  [ng/ml, median (quartile)] | 2.426  (1.765, 3.208) | 2.161  (1.726, 3.071) | 0.410 | 2.937  (1.706, 3.373) | 2.160  (1.747, 3.039) | 0.184 | 2.160  (1.729, 3.167) | 2.694  (1.807, 3.092) | 0.855 |
|  | Transferrin  [nmol/l, median (quartile)] | 0.163  (0.125, 0.218) | 0.145  (0.116, 0.198) | 0.234 | 0.171  (0.128, 0.217) | 0.146  (0.118, 0.200) | 0.275 | 0.155  (0.118, 0.202) | 0.168  (0.121, 0.210) | 0.729 |
|  | Lactoferrin  [ug/ml, median (quartile)] | 150.375  (72.531, 211.742) | 160.652  (122.807, 219.903) | 0.349 | 162.474  (77.170, 225.99) | 155.345  (90.257, 203.31) | 0.868 | 151.774  (80.687, 213.508) | 180.778  (103.192, 219.591) | 0.418 |
|  | IL-1β  [pg/ml, median(quartile)] | 16.370  (10.675, 21.262) | 14.190  (9.591, 22.031) | 0.427 | 15.284  (8.708, 21.953) | 14.658  (10.387, 20.873) | 0.609 | 14.783  (10.320, 20.873) | 17.259(9.440, 21.903) | 0.963 |
|  | IL-6  [pg/ml, median(quartile)] | 3.639  (1.996, 7.332) | 4.320  (1.670, 6.139) | 0.665 | 4.320  (2.086, 5.997) | 3.848  (1.738, 7.374) | 0.938 | 4.319  (1.882, 7.291) | 3.013  (1.572, 5.927) | 0.359 |
|  | PGE_2_  [pg/ml, median(quartile)] | 8.237  (4.659, 12.900) | 8.704  (3.874, 13.431) | 0.763 | 7.541  (3.221, 12.457) | 9.757  (4.678, 13.444) | 0.221 | 8.923  (4.985, 13.528) | 4.315  (2.817, 10.518) | 0.019 |
|  | H_2_O_2_  [mmol/L, median(quartile)] | 32.745  (26.233, 43.202) | 33.172  (24.215, 46.366) | 0.864 | 34.888  (24.692, 45.489) | 32.745  (25.874, 44.226) | 0.714 | 32.745  (25.522, 44.783) | 33.417  (25.357, 43.778) | 0.949 |
|  | NO  [mmol/L, median(quartile)] | 52.821  (33.333, 67.566) | 47.179  (34.837, 78.743) | 0.733 | 57.627  (40.678, 78.443) | 47.692  (33.690, 73.653) | 0.507 | 51.445  (33.611, 73.653) | 44.250  (37.725, 71.52) | 0.989 |
| **CSF** | Iron  [nmol/ml, median (quartile)] | 0.761  (0.393, 3.420) | 0.938  (0.473, 3.022) | 0.836 | 0.939  (0.393, 3.117) | 0.747  (0.406, 4.031) | 0.835 | 0.898  (0.414, 3.766) | 0.580  (0.365, 2.174) | 0.358 |
|  | Ferritin  [ng/ml, median (quartile)] | 2.543  (1.149, 11.042) | 2.736  (1.068, 11.851) | 0.659 | 2.179  (1.115, 10.835) | 2.943  (1.115, 11.851) | 0.758 | 2.458  (1.115, 11.042) | 2.943  (1.305, 12.096) | 0.448 |
|  | H-ferritin  [ng/ml, median (quartile)] | 1.129  (0.854, 1.560) | 1.147  (0.821, 1.665) | 1.000 | 1.142  (0.917, 1.397) | 1.158  (0.776, 1.661) | 0.994 | 1.145  (0.826, 1.584) | 1.096  (0.867, 1.631) | 0.881 |
|  | L-ferritin  [ng/ml, median (quartile)] | 1.319  (0.876, 1.671) | 1.306  (0.971, 1.792) | 0.819 | 1.38  (1.039, 1.595) | 1.302  (0.869, 1.707) | 0.674 | 1.319  (0.893, 1.673) | 1.310  (0.994, 1.676) | 0.522 |
|  | Transferrin  [nmol/l, median (quartile)] | 0.130  (0.106, 0.15) | 0.121  (0.101, 0.188) | 0.717 | 0.128  (0.103, 0.159) | 0.125  (0.105, 0.150) | 0.978 | 0.128  (0.106, 0.159) | 0.115  (0.095, 0.139) | 0.231 |
|  | Lactoferrin  [ug/ml, median (quartile)] | 106.825  (79.715, 163.372) | 100.553  (72.400, 162.942) | 0.595 | 103.741  (75.141, 192.896) | 101.919  (76.991, 157.002) | 0.882 | 99.119  (74.576, 163.372) | 114.008  (90.676, 156.884) | 0.283 |
|  | IL-1β  [pg/ml, median(quartile)] | 16.172  (10.267, 20.150) | 17.303  (10.481, 25.790) | 0.333 | 14.358  (9.241, 19.845) | 17.264  (10.467, 25.716) | 0.294 | 16.439  (10.259, 21.119) | 18.071  (11.378, 27.512) | 0.255 |
|  | IL-6  [pg/ml, median(quartile)] | 3.405  (1.933, 5.771) | 2.214  (1.662, 3.639) | 0.032 | 3.215  (1.828, 5.677) | 2.356  (1.666, 4.274) | 0.187 | 2.567  (1.802, 4.560) | 2.126  (1.586, 4.132) | 0.235 |
|  | PGE_2_  [pg/ml, median(quartile)] | 11.502  (5.771, 16.016) | 9.788  (4.540, 14.373) | 0.142 | 12.547  (6.215, 16.413) | 9.788  (5.491, 14.583) | 0.083 | 10.360  (5.771, 14.983) | 8.391  (4.397, 16.515) | 0.753 |
|  | H_2_O_2_  [mmol/L, median(quartile)] | 3.008  (1.973, 13.823) | 4.004  (2.019, 13.959) | 0.560 | 3.028  (1.973, 10.655) | 3.028  (2.019, 14.103) | 0.562 | 3.028  (1.973, 13.823) | 3.899  (2.031, 12.763) | 0.887 |
|  | NO  [mmol/L, median(quartile)] | 51.412  (36.158, 73.026) | 54.745  (38.505, 88.508) | 0.750 | 48.026  (34.872, 73.026) | 54.745  (38.922, 77.481) | 0.391 | 51.974  (38.686, 73.653) | 55.367  (34.884, 118.792) | 0.309 |

**Supplementary Table 3**. The levels of iron and its metabolism-related proteins in CSF and serum from control, PD-PIGD and PD-TD groups of drug-naive patients. P: Kruskal-Wallis test among control, PD-PIGD and PD-TD groups of drug-naive patients, α=0.008. P^1^: PD-PIGD group vs. Control group, α=0.017; P^2^: PD-TD group vs. Control group, α=0.017; P^3^: PD-TD group vs. PD-PIGD group, α=0.017.

|  | **Control group** | **PD-PIGD group** | **PD-TD group** | **P** | **P^1^** | **P^2^** | **P^3^** |
| --- | --- | --- | --- | --- | --- | --- | --- |
|  | **(35 cases)** | **(26 cases)** | **(40 cases)** |  |  |  |  |
| **CSF** |  |  |  |  |  |  |  |
| Iron  (nmol/ml, mean ± SD) | 0.478 ± 0.306 | 1.465 ± 1.581 | 4.840 ± 6.576 | **＜0.001** | 0.027 | **＜0.001** | **0.005** |
| Ferritin  (ng/ml, mean ± SD) | 7.336 ± 14.109 | 7.192 ± 7.907 | 3.784 ± 4.462 | 0.012 | **0.004** | 0.021 | 0.410 |
| H-ferritin  (ng/ml, mean ± SD) | 2.141 ± 0.730 | 1.450 ± 0.869 | 1.030 ± 0.687 | **＜0.001** | 0.090 | **＜0.001** | 0.18 |
| L-ferritin  (ng/ml, mean ± SD) | 2.496 ± 1.023 | 1.686 ± 0.647 | 1.117 ± 0.493 | **＜0.001** | 0.028 | **＜0.001** | **0.002** |
| Transferrin  (nmol/l, mean ± SD) | 0.067 ± 0.020 | 0.146 ± 0.086 | 0.135 ± 0.053 | **＜0.001** | **＜0.001** | **＜0.001** | 0.785 |
| Lactoferrin  (ug/ml, mean ± SD) | 67.444 ± 108.505 | 127.351 ± 66.859 | 109.148 ± 62.110 | **0.001** | **＜0.001** | **0.003** | 0.307 |
| **Serum** | | | | | | | |
| Iron  (nmol/m, mean ± SD) | 26.842 ± 27.517 | 2.919 ± 1.543 | 3.651 ± 1.510 | **＜0.001** | **＜0.001** | **＜0.001** | 0.163 |
| Ferritin  (ng/ml, mean ± SD) | 28.496 ± 33.413 | 26.315 ± 20.369 | 73.243 ± 57.233 | **0.002** | 0.441 | **0.001** | **0.011** |
| H-ferritin  (ng/ml, mean ± SD) | 41.451 ± 102.627 | 2.320 ± 1.053 | 2.363 ± 0.881 | **＜0.001** | **＜0.001** | **＜0.001** | 0.937 |
| L-ferritin  (ng/ml, mean ± SD) | 19.546 ± 19.514 | 2.304 ± 0.798 | 2.625 ± 0.960 | **＜0.001** | **＜0.001** | **＜0.001** | 0.326 |
| Transferrin  (nmol/l, mean ± SD) | 34.269 ± 50.260 | 0.153 ± 0.044 | 4.872 ± 17.119 | **＜0.001** | **＜0.001** | **＜0.001** | 0.078 |
| Lactoferrin  (ug/ml, mean ± SD) | 37.353 ± 48.528 | 166.232 ± 88.408 | 160.639 ± 70.891 | **＜0.001** | **＜0.001** | **＜0.001** | 0.880 |

|  | **Control group** | **PD-PIGD group** | **PD-TD group** | **P** | **P^1^** | **P^2^** | **P^3^** |
| --- | --- | --- | --- | --- | --- | --- | --- |
|  | **(35 cases)** | **(26 cases)** | **(40 cases)** |  |  |  |  |
| **CSF** |  |  |  |  |  |  |  |
| IL-1β  (pg/ml, mean ± SD) | 18.608 ± 16.714 | 18.210 ± 8.680 | 14.872 ± 8.519 | 0.186 |  |  |  |
| IL-6  (pg/ml, mean ± SD) | 1.413 ± 0.294 | 2.556 ± 2.139 | 4.308 ± 2.256 | **＜0.001** | 0.135 | **＜0.001** | **0.003** |
| PGE_2_  (pg/ml, mean ± SD) | 15.514 ± 16.394 | 11.758 ± 5.091 | 8.969 ± 4.930 | 0.103 |  |  |  |
| H_2_O_2_  (mmol/L, mean ± SD) | 4.497 ± 7.147 | 6.649 ± 6.611 | 11.340 ± 7.946 | **＜0.001** | 0.066 | **＜0.001** | 0.047 |
| NO  (mmol/L, mean ± SD) | 52.223 ± 30.709 | 66.781 ± 38.199 | 49.392 ± 22.392 | 0.182 |  |  |  |
| **serum** |  |  |  |  |  |  |  |
| IL-1β  (pg/ml, mean ± SD) | 43.749 ± 58.893 | 20.082 ± 14.330 | 19.547 ± 15.375 | 0.806 |  |  |  |
| IL-6  (pg/ml, mean ± SD) | 27.603 ± 34.399 | 3.407 ± 2.150 | 16.549 ± 35.500 | **＜0.001** | **＜0.001** | 0.130 | **0.002** |
| PGE_2_  (pg/ml, mean ± SD) | 39.685 ± 83.822 | 7.949 ± 4.696 | 13.595 ± 14.729 | 0.081 |  |  |  |
| H_2_O_2_  (mmol/L, mean ± SD) | 28.321 ± 37.262 | 39.907 ± 20.705 | 43.468 ± 35.546 | **0.008** | **0.007** | **0.005** | 0.880 |
| NO  (mmol/L, mean ± SD) | 30.101 ± 26.077 | 60.980 ± 34.380 | 48.375 ± 22.996 | **0.005** | **0.001** | 0.039 | 0.144 |

**Supplementary Table 4**. The levels of inflammatory factors in CSF and serum from control, PD-PIGD and PD-TD groups of drug-naive patients. P: Kruskal-Wallis test among control, PD-PIGD and PD-TD group of drug-naive patients, α=0.008. P^1^: PD-PIGD group vs. Control group, α=0.017; P^2^: PD-TD group vs. Control group, α=0.017; P^3^: PD-TD group vs. PD-PIGD group, α=0.017.
